# N-Acetylcysteine Protects Rats with Chronic Renal Failure from Gadolinium-Chelate Nephrotoxicity

**DOI:** 10.1371/journal.pone.0039528

**Published:** 2012-07-16

**Authors:** Leonardo Victor Barbosa Pereira, Maria Heloisa Massola Shimizu, Lina Paola Miranda Ruiz Rodrigues, Cláudia Costa Leite, Lúcia Andrade, Antonio Carlos Seguro

**Affiliations:** 1 Department of Nephrology, School of Medicine, University of São Paulo, São Paulo, Brazil; 2 Department of Radiology, School of Medicine, University of São Paulo, São Paulo, Brazil; University of Sao Paulo Medical School, Brazil

## Abstract

The aim of this study was to evaluate the effect of Gd-chelate on renal function, iron parameters and oxidative stress in rats with CRF and a possible protective effect of the antioxidant N-Acetylcysteine (NAC). Male Wistar rats were submitted to 5/6 nephrectomy (Nx) to induced CRF. An ionic - cyclic Gd (Gadoterate Meglumine) was administrated (1.5 mM/KgBW, intravenously) 21 days after Nx. Clearance studies were performed in 4 groups of anesthetized animals 48 hours following Gd- chelate administration: 1− Nx (n = 7); 2− Nx+NAC (n = 6); 3− Nx+Gd (n = 7); 4−Nx+NAC+Gd (4.8 g/L in drinking water), initiated 2 days before Gd-chelate administration and maintained during 4 days (n = 6). This group was compared with a control. We measured glomerular filtration rate, GFR (inulin clearance, ml/min/kg BW), proteinuria (mg/24 hs), serum iron (µg/dL); serum ferritin (ng/mL); transferrin saturation (%), TIBC (µg/dL) and TBARS (nmles/ml). Normal rats treated with the same dose of Gd-chelate presented similar GFR and proteinuria when compared with normal controls, indicating that at this dose Gd-chelate is not nephrotoxic to normal rats. Gd-chelate administration to Nx-rats results in a decrease of GFR and increased proteinuria associated with a decrease in TIBC, elevation of ferritin serum levels, transferrin oversaturation and plasmatic TBARS compared with Nx-rats. The prophylactic treatment with NAC reversed the decrease in GFR and the increase in proteinuria and all alterations in iron parameters and TBARS induced by Gd-chelate. NAC administration to Nx rat did not modify the inulin clearance and iron kinetics, indicating that the ameliorating effect of NAC was specific to Gd-chelate. These results suggest that NAC can prevent Gd-chelate nephrotoxicity in patients with chronic renal failure.

## Introduction

Gadolinium-chelate (Gd) agents are used because of its paramagnetic properties that increases the quality of images in magnetic resonance imaging (MRI) [1]. Despite the variety of Gadolinium based contrast, the pharmacokinetics is very similar. The compounds are water soluble, excreted unchanged by glomerular filtration, do not suffer biotransformation and have good distribution in the extracellular fluid with a rapid equilibrium between the intravascular and interstitial compartments. Notable exceptions to these rules include gadoxetic acid and gadofosveset trisodium. Gadoxetic acid is taken up by hepatocytes; up to 50% of the agent is excreted in feces and 50% in urine. The chelate is used for enhanced imaging of the liver. Between 80–96% of circulating gadofosveset trisodium is bound to plasma proteins, and the compound has been used as a blood pool agent [2].

The half life of Gd in patients with normal renal function is about 1 hour and 30 minutes and over 90% of the drug is excreted within 24 hours. Studies in patients with chronic renal failure (CRF) show the importance of renal excretion of gadolinium. Patients with kidney disease stage III (CrCL 31–60 ml/min) had half-life of excretion of gadolinium approximately 5.6 hours. Patients in stage IV (CrCL 15–30 ml/min) had half-life of 9.2 hours. In patients in stage V not on dialysis the elimination half-life of gadolinium-chelate can reach 34.3 hours [3]. Based on their chemical structure Gd contrast agents can be divided into 2 distinct classes: the linear and the macrocyclic agents, and based on their charge can be divided into the ionic and the non-ionic [3]. Some toxic effects of Gd based contrast agents are attributed to the chemical instability of the chelate and can lead to exchanges with other metals ions notably zinc, iron or calcium [4].

The macrocyclic agents are kinetically inert under physiologic conditions, in contrast, for the linear agents in vivo have a lower kinetic stability. On the other hand the nonionic compounds have a lower complex stability compared with ionic compounds. These physical and chemical characteristics of gadolinium-chelate are important because it has been demonstrated in vitro and in vivo that some cations in the body as Fe^3+^, Zn^2+^, and Cu^2+^ has the ability to displace the gadolinium its chelating exposing the metal in its free form raising toxicity. This phenomenon was called transmetallation.

Research in patients with chronic renal failure (CRF) shown that the use of gadolinium-chelate leads to a mobilization of iron body that can be analyzed by the kinetics of iron. In patients with CRF has been demonstrated that the application of gadolinium leads to a decrease in total binding capacity of iron (TIBC), increased serum ferritin and transferrin saturation [5]–[6]. Gadolinium-mobilized iron induced nephrogenic systemic fibrosis can be at least in part through induction of oxidative stress by Fenton reaction [5]–[6].

N-acetylcysteine (NAC) is antioxidant largely employed in the prevention of radiocontrast-induced renal failure; this effect is mediated by the suppression of oxidative stress induced renal tubular injury [7]–[9].

The purpose of the present study was to evaluate a protective effect of NAC on nephrotoxicity caused by Gadolinium chelates in rats with chronic renal failure.

## Materials and Methods

### Animals

Adult Male Wistar rats weighing between 180 and 210 *g*, were provided by the University of São Paulo School of Medicine for use in this study. Rats were anesthetized with intraperitoneal tribromoethanol 2.5% and were subject to either 5/6 nephrectomy, induced by right nephrectomy and ligation of two branches of the left renal artery. The surgery was made in one step. The animals were kept under standard laboratory conditions; standard water and food were provided ad libitum. Selected rats were treated with NAC (4.8 g/L) in drinking water, initiated 2 days before Gd administration.

### Tested Compounds

Gadolinium-chelate was approved and purchased from their respective manufacturer:

DOTAREM ® gadolinium-tetraazacyclododecanetetraacetic acid 0,5 mmol/ml (Gd-DOTA).

### Contrast Agent Administration

All rats except the normal group underwent 5/6 nephrectomy and were anesthetized, simulating a situation of risk for nephropathy related to radiological contrast. After 21 days of surgery, the animals were divided into five experimental groups.

Normal (n = 8)

Normal Group + Gd (n = 8) Gadolinium was injected once at a dose of 1.5 mmol/kg B.W. into the tail vein under anesthesia with intraperitoneal tribromoethanol 2.5%.

Nephrectomized (Nx):

Nx: 21 days after 5/6 nephrectomized rats + N-acetylcysteine (NAC) in drinking water (4.8 g/L) was started 2 days before clearance study (n = 6). This dose of NAC was the same employed in a previous study [8].

Nx + Gd - nephrectomized rats (n = 7) 21 days after nephrectomy, gadolinium was injected once at a dose of 1.5 mmol/kg body weight (b.w.) into the tail vein under anesthesia with intraperitoneal tribromoethanol 2.5%.

Nx+Gd+NAC - nephrectomized rats (n = 6) 21 days after nephrectomy, gadolinium was injected once at a dose of 1.5 mmol/kg b.w into the tail vein under anesthesia with intraperitoneal tribromoethanol 2.5%. N-acetylcysteine in drinking water (4.8 g/L) was started 4 days before clearance study.

The animals were placed in metabolic cages by 24 hours after injection of gadolinium in groups Nx, Nx+Gd, Nx+NAC and Nx+Gd+NAC. In metabolic cages, animals had free access to water and standard rat chow. The rats of Nx+NAC and Nx+Gd+NAC group received NAC in their cages. All groups remained in the metabolic cage for 24 hours. Urine was collected for determination of proteinuria (mg/24 h).

Urine collected in metabolic cage was used for measuring total urinary protein using the method of pyrogallol red. Proteinuria was determined using Sensiprot Labtest Diagnostica ®, through which the urine sample was mixed with a color reagent containing pyrogallol. The quantification of proteinuria was performed by spectrophotometric method based on the relationship between the absorbance of the sample and the standard solution (50 mg protein/dL).

### Blood Pressure

Before clearance studies, the animals were canulated with a PE- 60 in the carotid artery in order to measured Blood pressure by mercury manometer.

### Clearance Studies

To determine glomerular filtration rate, inulin clearance studies were performed. On the day of experiment, the animals were anaesthetized intraperitoneally with sodium thiopental (50 mg/kg B.W.). The trachea was cannulated with a PE-240 catheter, and spontaneous breathing was maintained. To control mean arterial pressure and allow blood sampling, a PE-60 catheter was inserted into the right carotid artery. For the infusion of inulin and fluids, another PE-60 catheter was inserted into the left jugular vein. In order to collect urine samples, a suprapubic incision was made, and the urinary bladder was cannulated with PE-240 catheter. After the surgical procedure had been completed, a loading dose of inulin (100 mg/kg B.W. diluted in 0.9% saline) was administered through the jugular vein. Subsequently, a constant infusion of inulin (10 mg/kg B.W.in 0.9% saline) was started and was continued at 0.04 ml/minute throughout the experiment. Three urine samples were obtained at the beginning and at the end of the experiment. Blood and urine inulin were determined using the anthrone method. GFR are expressed as ml/min/100 *g* B.W. At the end of clearance studies blood samples were collected from the catheter inserted in the carotid artery to analysis of the iron parameters and thiobarbituric acid reactive substances.

### Analysis of the Iron Parameters

To evaluate the effect of gadolinium on the parameters of the kinetics of iron were measured the Total Capacity Iron Binding (TBIC, µg/dL) and serum iron (µg/dL) in nonhemolyzed serum samples by spectrophotometric analysis using kits (Gold Analisa Diagnostica Ltda., Brazil). Transferrin saturarion was estimated from the ratio of serum iron to TBIC. Ferritin (ng/mL) was measured by immunoturbidimetric assay (Gold Analisa Diagnostica Ltda., Brazil).

### Reactive Oxygen Metabolites

Serum levels of thiobarbituric acid reactive substances (TBARS), which are markers of lipid peroxidation, were determined using the thiobarbituric acid assay. In brief, a 0.2-ml serum sample was diluted in 0.8 ml of distilled water. Immediately thereafter, 1 ml of 17.5% trichloroacetic acid was added. Following the addition of 1 ml of 0.6% thiobarbituric acid, pH 2, the sample was placed in a boiling water bath for 15 min, after which it was allowed to cool. Subsequently, 1 ml of 70% trichloroacetic acid was added, and the mixture was incubated for 20 min. The sample was then centrifuged for 15 min at 2000 rpm. The optical density of the supernatant was read at 534 nm against a reagent blank using a spectrophotometer. The quantity of TBARS was calculated using a molar extinction coefficient of 1.56×10^5^ M^−1^ cm^−1^.

### Statistical Analysis

Results were analyzed using ANOVA and the Student –Newman –Keuls *post hoc* test using GraphPad Prism (version 3.0). A *P* value <0.05 was considered statistically significant. Data are presented as mean ± SEM.

## Results

Normal rats treated with Gd presented a mean inulin clearance of 0.89±0.06 ml/min/100 g B.W, a value similar to untreated normal rats (0.82±0.05 ml/min/100 g B.W). The proteinuria was not different between the 2 groups: Normal rats (4.7±1 mg/24 hs) vs. Normal + Gd (5.9±1 mg/24 hs). The blood pressure was also not different between the 2 groups similar (normal  = 129±5 vs. normal + NAC  = 126±4 mmHg). These results indicate that at this dose Gd chelate is not nephrotoxic to normal rats.

The Nx+Gd group presented a higher proteinuria (21.5±3.8 mg/24 hs) than the Nx group (12.8±1.9 mg/24 hs, p<0.01). The proteinuria of NAC treated group was significantly lower (11.2±1.0 mg/24 hs) than Nx+Gd rats (p<0.01), a value not different from Nx group. In Nx+NAC group the proteinuria was 10.7±1.5 mg/24 hs.

Blood Pressure (BP) was measured and showed no statistical difference between the 4 groups (Nx  = 163±8; Nx + NAC  = 161±12; Nx+Gd = 170±8 and Nx+Gd+NAC = 151±12 mmHg). Mean dose of NAC ingestion in Nx+Gd+NAC was 72.7±10.3 mg/day.

As we can see in Table 1, 48-hs after Gd administration the inulin clearance was significantly lower in Nx+Gd rats (0.25±0.03 ml/min/100 g B.W p<0.01) when compared with Nx-group (0.40±0.03 ml/min/100 g B.W). NAC prevented the decreased of glomerular filtration in Nx-rats treated with Gd (0.41±0.07 ml/min/100 g B.W). NAC administration to Nx rat did not modify the inulin clearance.

**Table 1:** Inulin clearance and iron parameters in Nx (nephrectomized); Nx + Gd (nephrectomized plus Gadolinium chelate); Nx Gd + NAC (nephrectomized plus Gadolinium chelate and N-acetylcysteine); Nx+NAC (nephrectomized plus N-acetylcysteine).

Groups	Inulin clearance (ml/min/100 g)	TIBC (µg/dl)	Transferrin saturation (%)	Ferritin (ng/ml)	Serum iron (µg/dl)
Nx	0.40±0.03	225±10	25.2±2.7	26.3±2.7	65±11
Nx+NAC	0.36±0.02	230±18	26.7±2.4	30.2±4.1	60±4
Nx+Gd	0.25±0.03^b^	172±12^b^	51.6±5.0^a^	47.7±7.2^b^	87±7
Nx+Gd+NAC	0.41±0.07^d^	232±10^d^	32.2±5.6^e^	28.6±3.1^d^	76±15

Data are mean ± s.e.m.

a p<0.001

b p<0.01

c p<0.05 vs Nx.

d p<0.05 ^e^p<0.01 vs Nx+ Gd.

TIBC (total iron binding capacity).

The ferritin levels were significantly higher in Nx+Gd group (47.7±7.2 ng/dl) than in Nx group (26.3±2.5 ng/dl, p<0.05).The group Nx+Gd+NAC showed a serum ferritin level of 28.6±1.3 ng/dl, similar to group Nx and lower than group Nx+Gd (p<0.05).

The transferrin saturation in Nx+Gd group was significantly higher than the group Nx (Nx+Gd = 51.6±5.0% vs. Nx  = 25.2±2.7%, p<0.01). The group Nx + NAC+ Gd showed a transferring saturation 32.2±5.6% similar to the groups Nx and Nx+NAC, and lower than group Nx+Gd (p<0.01).

Total iron binding capacity (TIBC) in Nx+Gd group (172±12 µg/dl) was lower than Nx group (225±10 µg/dl p<0.01). The group Nx + Gd NAC showed a mean TIBC of 232±10 µg/dl a value similar to Nx group and larger than Nx+Gd group (p<0.01). Serum iron was not statistically different between the 4 groups.

**Figure 1:**
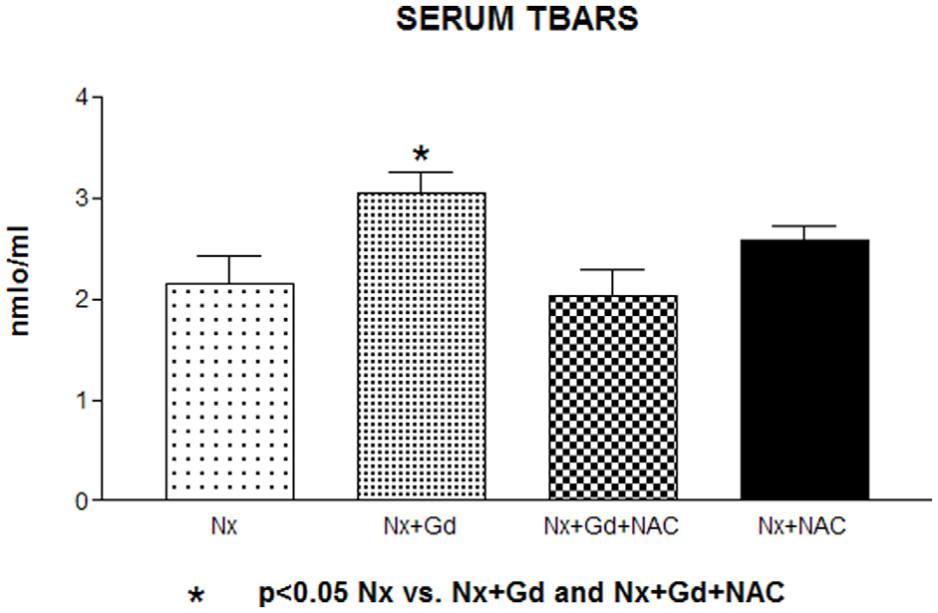
Serum thiobarbituric acid reactive substances in groups: Nx (nephrectomized); Nx + Gd (nephrectomized plus Gadolinium chelate); Nx Gd + NAC (nephrectomized plus Gadolinium chelate and N-acetylcysteine); Nx+NAC (nephrectomized plus N-acetylcysteine). p<0.05 Nx vs. Nx + Gd and Nx+Gd+ NAC.

The mean serum TBARS of Nx+Gd rats was significantly higher (3.04±0.2 nmol/mL) than in Nx group (2.14±0.28 nmol/mL, p<0.05) (Fig. 1). NAC pretreatment restored the TBARS levels to a value not significantly different from Nx (2.04±0.24 nmol/mL). These data suggest that Gd chelate increases oxidative stress in Nx rats and that the pretreatment of Nx+Gd rats with NAC did not increased this parameter.

## Discussion

Our results demonstrate that nephrectomized 5/6 (Nx) animals submitted to a single injection of gadolinium, showed 48 hours after contrast administration, a significant decrease in GFR (evaluated by the gold standard method, the inulin clearance) and increased proteinuria rate, when compared with the 5/6 Nx rats which did not receive Gd chelate. Furthermore, we observed in nephrectomized rats that received gadolinium (Nx + Gd) significant changes in the parameters of iron metabolism, ferritin, transferrin saturation and a decrease in total iron binding capacity (TIBC) compared to nephrectomized rats (Nx) with statistical significance.

The administration of NAC to Nx rats which received Gd prevented the fall in GFR and the increase of proteinuria with gadolinium and restored the kinetic parameters of iron to values similar to the Nx group. These effects were not due to a possible effect of NAC on Nx, since all parameters measured in Nx+NAC group were not different from the Nx group.

The ameliorating effect of NAC was specific to the Nx + Gd group, since all parameters were not improved by NAC in Nx+NAC group.

Recent studies, including in humans, have demonstrated the potential nephrotoxicity of gadolinium [10]. Erley et al published a prospective randomized study in patients with serum creatinine >1.5 mg/dl, showing a decrease in GFR of around 50% of patients exposed to gadolinium compared with 45% in patients who received iodinated contrast [11]. Briguori et al in a retrospective study which included a great number of patients reported an incidence of contrast nephropathy associated to administration of iodinated contrast agents and gadolinium (Gadodiamide and Omniscan) dilutes with iso-osmolar iodinated contrast media in 3∶ 1 parts, with around 28% compared to 6.5% in patients who receiving only iodinated contrast [12]. Rapid deterioration of renal function and need of hemodialysis after Gd administration was recently related in a diabetic patient with CRF [13]. A recent study of the use of Gd for arterial interventions related that the highest risk for the development of acute renal failure occurred in patients with serum creatinine levels higher than 3.0 mg/dL and in those receiving more than 0.4 mmol/Kg of Gd [14].

For many years, there was no evidence of the precise mechanism of toxicity of Gd contrast agents until the emergence of nephrogenic systemic fibrosis. A report from Swaminathan S et al about the pathophysiology of this disorder suggested the involvement of oxidative stress mediated by the phenomenon of the transmetallation of gadolinium as a mechanism responsible for its toxicity. The transmetallation as discussed above refers to the ability of other cations of the organism (zinc, iron, cooper and calcium) to displace the gadolinium from its chelate and release it in free form [5].

Swaminathan S et al demonstrated that administration of gadolinium-chelate in patients with CRF who developed nephrogenic systemic fibrosis (NSF) results in marked changes in iron metabolism parameters with decreased TIBC, increased transferrin saturation and serum ferritin with consequent mobilization of body iron. They noted that beyond the CRF, patients who developed NSF were using high doses of erythropoietin, increased loads of body iron, elevated parathyroid hormone (PTH) and elevated levels of systemic markers of inflammation, as cumulative risk factors for the development of NSF [5]–[6].

The iron mobilized, as well as gadolinium in its free form, can be toxic to tissues through the induction of oxidative stress mediated by the Fenton reaction. So the latest evidence shows that a combination of free gadolinium, iron released by Fenton reaction, oxidative stress and inflammatory factors were possibly involved in the pathogenesis of nephrotoxicity related to gadolinium and the emergence of NSF.

All these findings indicate that studies are necessary to the prevention of Gadolinium-chelate nephrotoxicity and to nephrogenic systemic fibrosis in patients with chronic renal failure [15].

The use of NAC in our study was justified by its ability to decrease the oxidative stress generated during events of cellular stress [16]–[18]. N- acetylcysteine (NAC) is an antioxidant thiol that can enter to the chain of glutathione synthesis and serves as a source of sulfhydryl groups for the cells, acting as a scavenger of reactive oxygen species (ROS).

TBARS is an indirect method to evaluate oxidative stress, it represents the interaction between reactive oxidant radicals with the lipid membrane of the cell. Previous studies have demonstrated that TBARS levels are increased in urine of rats with CRF [17] and in urine and plasma of patients with chronic renal failure [19]–[20] and in adult primary graft recipients of deceased renal donors [21].

In the present study, TBARS increased in Nx+Gd rats suggesting an increase in oxidative stress induced by Gd. NAC administration restored TBARS levels to a value similar to Nx-rats indicating an antioxidant effect of NAC in this group.

As already expected, we did not find skin lesions in rats studied because we use a relatively low single dose of macrocyclic gadolinium-chelate in comparison to recent studies which used linear and nonionic compounds several days in rats with chronic kidney disease [22]. We studied the animals 48 hours after contrast administration because Brillet at al demonstrated that Gd-DTPA increased serum creatinine to a greater value 24 and 48 hs after administration [23].

The mechanism of kidney toxicity of Dotaren, although thought to be related to hyperosmolarity, is not entirely understood. High osmolar radiocontrast media (sodium diatrizoate) or an equivalent volume of isoosmolar manitol (1,100 mOsm/L) were administered in rats with chronic NO depletion induced by pretreatment with L-NAME during 8 weeks [24]. Radiocontrast media application induced a significant decline in glomerular filtration rate (inulin clearance) in L-NAME hypertensive rats whereas no effect of were observed in mannitol-infused L-NAME hypertensive rats. Heyman SN et al demonstrated that iothalamate (an ionic high osmolar agent), ioxoglate (low osmolar agent) and iohexol (nonionic agent) produced a rise in plasma endothelin (vasoconstrictor) and induced a reduction n renal blood flow [25]. The rise in plasma endothelin was not correlated with postinjection plasma osmolality. In the same study plasma endothelin did not increase after injection of hypertonic saline (2,590 mOsm/kg), glucose (2,560 mOsm/kg) and mannitol (1,510 mOsm/kg) and the release of endothelin from cultured endothelial cells increased with the addition of iothalamate and iohexol, while did not change with the addition of hypertonic saline and hypertonic glucose. These experiments demonstrate that the decrease in GFR was independently of osmotic load.

Since DOTA is excreted by glomerular filtration and the GFR of the rat corrected by body weight is 5–6 fold higher than man, we used an elevated dose of Gd-DOTA [26]. Doses of Gd-DOTA up to 0.325 mL/Kg and even uncommon greater than 0.2 mM/Kg were employed in patients [27].

We believe that our main result was the ability of NAC to reverse the decline in GFR and the increase in proteinuria in rats with CRF receiving gadolinium and prevent the mobilization of body iron by the Fenton-transmetallation reaction. The antioxidant effect of NAC can prevent gadolinium toxicity and even the development of nephrogenic systemic fibrosis.

Future studies in patients are necessary to prove this beneficial effect of NAC in Gd nephrotoxicity.
